# Managing Sleep Disturbances in Cirrhosis

**DOI:** 10.1155/2016/6576812

**Published:** 2016-05-03

**Authors:** Xun Zhao, Philip Wong

**Affiliations:** Department of Gastroenterology and Hepatology, Royal Victoria Hospital, McGill University, 1001 Boulevard Decarie, Montreal, QC, Canada H4A 3J1

## Abstract

Sleep disturbances, particularly daytime sleepiness and insomnia, are common problems reported by patients suffering from liver cirrhosis. Poor sleep negatively impacts patients' quality of life and cognitive functions and increases mortality. Although sleep disturbances can be an early sign of hepatic encephalopathy (HE), many patients without HE still complain of poor quality sleep. The pathophysiology of these disturbances is not fully understood but is believed to be linked to impaired hepatic melatonin metabolism. This paper provides an overview for the clinician of common comorbidities contributing to poor sleep in patients with liver disease, mainly restless leg syndrome and obstructive sleep apnea. It discusses nondrug and pharmacologic treatment options in these patients, such as the use of light therapy and histamine (H1) blockers.

## 1. Introduction

Patients with liver cirrhosis report significantly more sleep disturbances than healthy individuals [1–3]. Common complaints in regard to sleep are prolonged time to fall asleep (longer sleep latency), shortened sleep duration, daytime sleepiness (somnolence), poor sleep quality, and frequent nocturnal awakenings [3–6]. Case control studies report that insomnia and daytime sleepiness are the most commonly reported problems. Insomnia in patients with hepatic cirrhosis varies from 26% to 42% while less than 10% of the control groups of healthy individuals typically experience insomnia [7]. Recent studies have even shown quantitative evidence of sleep disturbances in cirrhosis patients, which when compared to controls have reduced REM sleep on polysomnography [4].

Hepatic encephalopathy (HE) is classified as either overt HE or covert HE as per recent published guidelines [8]. Covert HE is a new term which is created by grouping patients with minimal HE and West Haven Criteria (WHC) Grade I HE together. Minimal HE patients often appear and act normal and require specialized psychometric testing to detect the underlying HE. For this reason, they are often underdiagnosed and do not benefit from early interventions. Estimating HE grade in patients with West Haven Criteria (WHC) grade I is often subjective between different physicians, and they often disagree in the HE severity. Clinically obvious HE, WHC grades 2–4, are now classified as overt HE and do not usually require specialized testing. It is hoped that these new classifications of covert HE and overt HE will lead to a more standardized approach to these patients, in both their classification and their management.

Covert HE is estimated to affect up to 80% of patients with either compensated or decompensated cirrhosis [9]. A poor quality of life (QOL), including sleep disturbances, is a common complaint in these patients [10], as well as more frequent falls and injuries, motor vehicle accidents, and decreased survival [11]. Because specific management guidelines exist [8], it is important to identify those individuals affected with covert HE and treat them separately from cirrhotic patients without HE who are afflicted with another disorder. Although conventional psychometric tests are still commonly used, these traditionally paper-pencil tests (i.e., Psychometric Hepatic Encephalopathy Score, PHES) are more efficiently applied by a trained psychologist, are time consuming, and, in some cases, have associated proprietary costs with their use. Conversely, the availability of free, downloadable tools (apps) such as the Inhibitory Control Test (ICT) or the Encephalapp (i.e., Stroop test) has allowed inexpensive, rapid, simple screening methods with high sensitivity and specificity to be administered by the clinician without the need for trained personnel [12–14].

Despite the fact that disturbance in sleep rhythm is one of the earliest signs of covert hepatic encephalopathy, there has been no correlation found between the degree of sleep disturbance and the presence of hepatic encephalopathy. There may, however, be a relationship between daytime sleepiness and the development of HE [7]. In primary biliary cirrhosis, excessive daytime sleepiness has been shown to worsen symptoms of fatigue [6], which in turn increases mortality, in particular cardiac death [15–17]. Furthermore, sleep disturbances cause heavy losses to a patients' quality of life [18–20] and perhaps produce loss of cognitive functions [21] or depression [22]. Better quality of life has been shown to decrease mortality independently of the MELD score [20].

## 2. Material and Methods

Internet data bases used were PubMed and Google Scholar. The following search words were used: Sleep disturbance Cirrhosis. The information of 55 references was cited in this review article.

## 3. Review of Literature and Discussion

### 3.1. Pathophysiology

The pathophysiology of disturbances in sleep-wake cycles in liver cirrhosis is not well understood. Earlier studies have shown that patients suffering from liver cirrhosis have higher melatonin levels throughout the day and delayed onset of melatonin peaking during the night [23]. This disturbance in melatonin has been correlated to delayed sleep onset [24]. The melatonin response to light also seems to be reduced in cirrhosis [25] and is likely due to impairment of hepatic metabolism of melatonin. However, these melatonin models currently only correlate weakly with symptoms and cannot fully explain the well-documented poor sleep quality in cirrhotic patients.

### 3.2. Common Comorbidities

Obstructive sleep apnea (OSA) is found more commonly in patients with liver cirrhosis [26, 27]. Patients with OSA have an over-4-times higher prevalence of liver cirrhosis as compared to the general population [26]. Furthermore, the use of daytime sleepiness questionnaires found no differences in symptoms between patients with OSA and cirrhosis versus patients with OSA alone [28]. Thus, it suggests that a comorbid obstructive sleep apnea should often be considered when a patient with liver cirrhosis complains of fatigue and sleep disturbances. Bajaj et al. in a small sample size study of 17 patients found improvement in executive functions after CPAP treatment of OSA, which was in turn an independent predictor of concomitant liver cirrhosis [29]. As it is becoming clear that OSA is an* independent* risk factor of liver injury [30–33], the early detection and initiation of treatment of sleep apnea now play a pivotal role in the management of hepatic cirrhosis. Restless leg syndrome is more commonly reported in patients with cirrhosis and sleep disturbances than the general population [34], although the evidence is still unclear as to whether it would be a treatable cause of fatigue and sleep disturbance [35, 36].

Pruritus is another common symptom of chronic cholestatic liver disease. As a result, treatment of pruritus may improve sleep disorders associated with liver cirrhosis. A placebo controlled study [37] showed that naltrexone not only improves daytime itching but also decreases sleep disturbance as assessed by visual analogue scores when compared to the placebo group (42 ± 9 versus 22 ± 16 in change in VAS score (*p* < 0.004)). Thus, controlling pruritus may be another important target in the management of cirrhotic patients complaining of sleep disturbances.

### 3.3. Nonpharmacological Treatments

Treatment options for these patients can be divided into nonpharmacological and pharmacological choices. Given that there is evidence of circadian deregulation and delayed melatonin response in cirrhotic patients as previously mentioned, parallels could be drawn to delayed sleep phase syndrome (DSPS), which has a similar pathophysiology [38, 39]. Light therapy has been shown to be effective in DSPS [24, 38]. Very few studies have looked at effectiveness of administering bright light in the morning in an attempt to synchronize daytime melatonin levels in liver cirrhosis patients and decrease daytime sleepiness [40]. A small randomized control trial of 12 cirrhotic patients showed no obvious beneficial effect, if any, to administration of bright light therapy in terms of sleep onset, quality, and daytime sleepiness [40]. Supportive evidence for light therapy being an effective treatment for sleep disturbance rests mainly on case reports [41].

Late evening snacking by patients with cirrhosis is a potential intervention to delay sarcopenia [42, 43], by capitalizing on all anabolic periods during the day. Patients should be counseled on the potential increased risk of acid reflux and potential worsening of any preexisting sleep disturbances [42–44]. Proper planning of an evening snack by allotting at least 1-2 hours after eating and before lying supine will be helpful to prevent this complication. Late evening snacking has also been shown to be more prevalent in NAFLD (nonalcoholic fatty liver disease), and insulin-resistance significantly correlates with daytime sleepiness [45]. Nonetheless, no studies have looked directly at the question as to whether late evening snacking directly correlates with daytime sleepiness in liver cirrhosis.

### 3.4. Pharmacological Treatments

Several pharmacologic agents have been tried. A double blind randomized placebo-control trial of 35 patients with biopsy proven liver cirrhosis has shown that after a 10-day regimen of 25 mg hydroxyzine, 30% of placebo versus 65% of treatment group had a sleep efficiency increase greater than 30% (*p* < 0.04) [46]. Sleep efficiency was defined as total time spent in bed for sleeping purposes divided by the difference of bedtime and wake-up time. There was also a decrease in nighttime activity (defined by the difference of bedtime and wake-up time) in hydroxyzine-treated patients as compared to placebo (−36% versus +16%, *p* < 0.015). There was no significant difference in total daytime activity between the two groups and the study did not address the issue of daytime sleepiness. However, a visual assessment score of having subjects grading their quality of sleep on a scare from 1 to 10 showed a 40% improvement in the hydroxyzine-treated group versus 0% in the placebo group (*p* < 0.04). It is important to note that the trial was of short duration, only 10 days, and did not hypothesize on the possible long-term benefit of histamine H1 blockers. Although one patient had to discontinue hydroxyzine upon development of hepatic encephalopathy (HE), it is unclear in their report whether this episode of HE can be firmly attributed to this small dose of hydroxyzine versus other causes.

Lactulose is an effective treatment for hepatic encephalopathy. A randomized controlled trial has shown significant improvement after 3 months in subjective response to sleep/rest categories of the SIP (sickness impact profile) questionnaire in patients with hepatic encephalopathy treated with lactulose as compared to those who did not (*p* < 0.013) [47]. It is important to note that these improvements are shown in patients with minimal hepatic encephalopathy and did not apply to all patients with liver cirrhosis.

Primary biliary cirrhosis (PBC) is an autoimmune liver disease in which patients commonly report fatigue as part of their illness [48] and several studies have looked at pharmacological interventions for these symptoms. Treatment of fatigue in PBC with fluvoxamine [49], fluoxetine [50], ondansetron [51], and several other medications has been unsuccessful. However, a recent study with modafinil showed a decrease in daytime somnolence in 31 of 42 patients that were given 3-day regiment of that medication [52]. Another study with modafinil looked at improvement in daytime somnolence in PBC via the well-established Epworth Sleepiness Scale; it showed that the pretreatment ESS is 15 ± 3.3, which fell to 8 ± 6 posttreatments (*p* < 0.0005) [53]. Perhaps modafinil could be effective combating daytime sleepiness in other causes of liver cirrhosis.

## 4. Conclusion

Sleep disturbances are a common problem in liver cirrhosis. However, adequate management for these debilitating symptoms has been studied in very little depth and remains an important unaddressed concern for these patients. A reasonable approach to patients with known cirrhosis complaining of poor sleep quality or daytime sleepiness is to first and foremost rule out the presence of HE, such as West Haven Criteria Grade 1 or minimal HE (covert HE). Secondly, as obstructive sleep apnea is often a comorbid condition in liver cirrhosis, its presentation or contribution to daytime sleepiness is virtually indistinguishable from that of liver cirrhosis associated daytime sleepiness. The management of OSA is effective and well-studied, and if they have other associated comorbidities such as restless leg syndrome, treatment of these complications can only help in resolving the patient's sleep disturbance.

An underutilized, nondrug approach to sleep disturbances is light therapy. Its lack of toxicity and “natural” approach would likely appeal to most patients with cirrhosis, who often suffer from “pill burden,” having to take multiple medications. Its utility should be validated in larger studies. But low dose antihistamines such as hydroxyzine, although appearing very promising, must be used with caution over the risk of precipitating hepatic encephalopathy, and these patients need closer monitoring in the short term until verified safe. Lactulose may have some documented benefit on sleep quality and must be studied in more detail. Lastly, use of the stimulant modafinil was effective in combating daytime somnolence in primary biliary cirrhosis (PBC), and perhaps it can be useful if applied to other conditions causing liver cirrhosis. Pharmacotherapy remains limited in scope and efficacy, and its application remains on a case-by-case basis in cirrhotic patients with sleep disturbances.
